# Expectation of a Decrease in Pain Affects the Prognosis of Pain in Cancer Patients: a Prospective Cohort Study of Response to Morphine

**DOI:** 10.1007/s12529-017-9644-5

**Published:** 2017-03-06

**Authors:** Hiromichi Matsuoka, Kazuhiro Yoshiuchi, Atsuko Koyama, Chihiro Makimura, Yoshihiko Fujita, Junji Tsurutani, Kiyohiro Sakai, Ryo Sakamoto, Kazuto Nishio, Kazuhiko Nakagawa

**Affiliations:** 1 Department of Psychosomatic Medicine, Kindai University Faculty of Medicine, 377-2 Ohno-Higashi, Osaka-Sayama, Osaka 589-8511 Japan; 2 Department of Stress Sciences and Psychosomatic Medicine, Graduate School of Medicine, The University of Tokyo, Tokyo, Japan; 3 Department of Genome Biology, Kindai University Faculty of Medicine, Osaka, Japan; 4 Department of Medical Oncology, Kindai University Faculty of Medicine, Osaka, Japan

**Keywords:** Cancer pain, Expectation in pain decrease, Morphine treatment, Psycho-oncology

## Abstract

**Purpose:**

Cancer pain is a multidimensional experience that includes physiological, sensory, affective, cognitive, behavioral, and sociocultural dimensions. Few prospective studies have examined the relationship between a patient’s expectation of pain improvement and the pain prognosis. The aim of this prospective study was to investigate whether patients’ expectation to pain reduction was associated with pain intensity after morphine treatment in opioid treatment-naïve patients with various types of cancer**.**

**Methods:**

The subjects were patients scheduled for cancer pain treatment with morphine who were taking nonsteroidal anti-inflammatory drugs daily. Morphine treatment was performed according to the standard method, including titration (NCCN Guidelines™, Adult Cancer Pain). Simple regression analysis was performed between pain intensity numerical rating scale (NRS) (day 8) as the dependent variable, expectation of pain decrease NRS (day 1), tumor types, and the following covariates as independent variables: patients’ characteristics such as age, gender, PS (day 1), genotype of catechol-O-methyltransferase, total scores of Hospital Anxiety and Depression Scale (day 1), and pain intensity NRS (day 1). Multiple regression analysis was performed using forced entry methods with pain intensity NRS (day 8) as the dependent variable, and expectation of pain decrease NRS (day 1) and the covariates as independent variables that had a p value <0.05 in the simple regression models.

**Results:**

A total of 100 patients with baseline data were included, and 97 patients (51% female) met the inclusion criteria. Patients with a high expectation of pain decrease NRS had a significantly lower pain intensity NRS (day 8) (p = 0.001).

**Conclusion:**

Non-pharmacological factors such as expectations for pain treatment could also be important factors to treat cancer pain, which might be associated with communication skills in physicians.

## Introduction

Pain is a common symptom in cancer patients [1, 2] that increases in prevalence and intensity with disease progression [3, 4] and influences multiple domains of quality of life [5–8]. Ahles et al. [9] and McGuire et al. [10] have described cancer pain as a multidimensional experience, including physiologic, sensory, affective, cognitive, behavioral, and sociocultural dimensions.

There is a need for a multifaceted approach to non-cancer chronic pain that includes the effects of catastrophizing (exaggeration of pain) [11] and expectation of pain improvement [12, 13]. These factors are related to pain severity, disorder, and prognosis and provide important information for psychosomatic treatment of non-cancer chronic pain [14]. Studies in patients with whiplash injury due to traffic accidents and in those with back pain have shown positive correlations between expectation of pain improvement and a decrease in actual pain [12, 13]. There are several studies investigating the effect of expectation as a predictor for pain level in patients with non-specific low back pain and total joint arthroplasty [15, 16], and according to the systematic review, 15 of the 16 studies showed that positive expectations were associated with better health outcomes including pain [17].

Furthermore, almost all guidelines have mentioned the importance not only the use of pharmacological therapy (especially in chronic opioid therapy) but also the non-pharmacological therapy including reduction of pain catastrophizing and rise of expectation of patients in chronic non-cancer pain because safe and effective chronic opioid therapy for chronic non-cancer pain requires clinical skills and knowledge in both the principles of opioid prescribing and on the assessment and management of risks associated with opioid abuse, addiction, and diversion [18, 19].

On the other hand, in treating cancer patients, the main approaches to cancer pain (= physical pain) are drug therapies, such as non-steroidal anti-inflammatory drugs (NSAIDs) and opioids. Since the report is less about the importance of non-drug therapies (for example, interview that give hope to the patient), which are often frequently used in chronic pain models of non-cancer, results and guidelines of non-cancer patients study [18, 19] might not be used as they are. Knowing expectation influence on the pain prognosis, we believe that personalized medicine, which predicted the pain prognosis, might be spread in the field of treatment of cancer pain. It may also be possible to treat cancer patients of chemical coping, which has recently become a problem [20–22].

However, there are few studies on expectation of symptom improvement before treatment in cancer patients [23], and the relationship between a patient’s expectation of pain improvement and the pain prognosis has not been examined in cancer patients.

In this prospective study, we investigated whether patients’ expectation to pain reduction was associated with pain intensity after morphine treatment in a cohort of opioid treatment-naïve patients with various types of cancer.

## Materials and Methods

### Patients and Samples

**Fig. 1:**
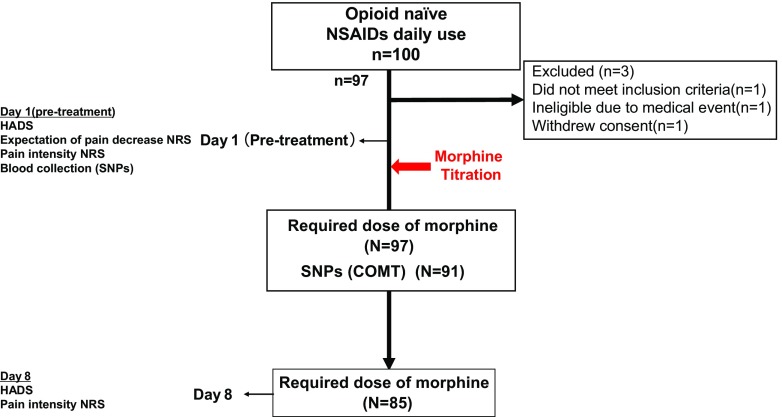
Flowchart of the study. A total of 100 patients with baseline data were included, 3 patients were ineligible, 1 patient did not meet inclusion criteria, 1 patient was due to medical event, and 1 patient withdrew consent. All 97 patients underwent gene expression analysis. Ninety-one patients were evaluated in genotype analysis. The required dose of morphine (day 8) was evaluated in 85 patients. Assessments were conducted day 1 (pre-treatment) and on day 8 (8 week after treatment). *NSAIDs* non-steroidal anti-inflammatory drugs, *NRS* Numerical Rating Scale, *SNPs* single nucleotide polymorphisms, *COMT* catechol-*O*-methyltransferase

This prospective study was conducted from 2009 to 2012 at the Kindai University Faculty of Medicine and Sakai Hospital. A total of 100 patients with opioid treatment-naïve and histologically confirmed malignant neoplasms who were scheduled to undergo opioid treatment were evaluated in the study. They were recruited and selected by non-probabilistic convenience sampling by their physician’s selections from the outpatient and inpatient service of Department of Medical Oncology. Of these patients, 97 met inclusion criteria of ≥18 years of age, a verified cancer diagnosis, cancer pain suitable for morphine treatment (excluding neuropathic pain, predominant incidental pain, glomerular filtration rate <30 ml/min, no history of opioid/drug abuse or alcoholism from self-reported questionnaire), daily use of non-steroidal anti-inflammatory drugs, and ability to give signed informed consent. Three patients were ineligible, 1 patient did not meet inclusion criteria, 1 patient was due to medical event, and 1 patient withdrew consent. All 97 patients underwent gene expression analysis, but only 91 patients were evaluated in genotype analysis because the DNA samples were insufficient in 6 cases. The required dose of morphine (day 8) was evaluated in 85 patients, excluding 12 who could not continuously receive morphine due to adverse effects or death (Fig. 1).

Clinical features including age, sex, Eastern Cooperative Oncology Group (ECOG) performance status (PS), and types of primary malignant neoplasm were recorded. Morphine treatment was performed according to the standard method, including titration (NCCN Guidelines™, Adult Cancer Pain) by specialized palliative care doctors. On this step, they explained standardized information about potential benefits and adverse effects to their patients. Assessments were conducted pre-treatment (day 1) and 1 week after treatment (day 8).

This study was approved by the Regional Committee for Medical Research Ethics, Kindai University Faculty of Medicine and informed consent was obtained from all individual participants included in the study.

### Measures

#### Pain Score

Pain score was assessed with 0–10 Numerical Rating Scale (NRS, 0 = no pain to 10 = the worst imaginable pain) (pain intensity NRS) on day 1 (pre-treatment) and on day 8 (1 week after treatment) by asking patients by the following question; “How intense was your average pain for the past 24 hours?”

### Patient’s Expectation of a Decrease in Pain

Patients’ expectation of a decrease in pain determined via a questionnaire with a 0–10 NRS (0 = no improvement to 10 = full improvement) on day 1 (pre-treatment) (expectation of pain decrease NRS (day 1)) and on day 8 (1 week after treatment). Nurses who were not related to this study handed questionnaires to patients. The questionnaire had the following question; “How much do you think your pain will get better?” The questionnaire was self-administered.

### Hospital Anxiety and Depression Scale

To assess correlations with pain intensity NRS (day 1 and day 8), anxiety/depression before treatment (day 1) and on day 8 was evaluated using the Hospital Anxiety and Depression Scale (HADS). In accordance with a systematic review by Cosco et al. [24], we use HADS total scale rather than the subscales.

### Genotyping

The single nucleotide polymorphisms (SNPs) of catechol-*O*-methyltransferase (COMT) 472G→A (rs4680, p.Val158Met) also modulate the genetic response to opioid medications [25]. We performed an exploratory study in the first half of 50 cases at the beginning. To find predictive biomarkers of the treatment outcome of morphine, we performed a functional genotype analysis of *OPRM1* 118A→G (rs1799971, p.Asn40Asp) and *COMT* 472G→A (rs4680, p.Val158Met). The treatment outcome of morphine was examined based on the plasma concentration of morphine and the required dose according to genotype on days 1, 2, and 8 [26]. On day 1, blood sample for genotyping was conducted, and measures (pain score, HADS, and patients’ expectation) were conducted on day 1 (before and post treatment), day 2, and day 8. After that, we performed a confirmatory study in the second half of 50 cases. At this time, we measured only COMT, which was a significant difference in the exploratory study. Since this was not related to the results of the present study and we performed same psychological measures, we have decided to treat all patients as one group and omit details on day 1 (post treatment), day 2, and blood sampling except COMT genotypes on the Fig. 1 in order to avoid confusion.

Genomic DNA isolated from blood samples using a QIAamp® DNA Blood Mini Kit (Qiagen) was amplified with the following primers: *COMT* forward, 5′-GAT TCA GGA GCA CCA GCC CTC C-3′ and reverse (intronic), 5′-CAC TGA GGG GCC TGG TGA TAG TG-3′. PCR was performed in a 20-μl volume containing 20 ng of template, 0.5 μM of each primer, Ampdirect Plus (Shimadzu Corp., Kyoto, Japan), and 0.5 units of NovaTaq™ DNA Polymerase (Merck, Darmstadt, Germany). Amplification was performed for 35 cycles (95 °C for 30 s, 60 °C for 30 s, and 72 °C for 45 s). The resulting PCR fragments consisting of 210 bp (*COMT*) were directly sequenced with the corresponding forward and reverse primers, respectively [26].

### Statistical Analysis

All data are expressed as mean ± standard deviation. We first undertook a bivariate relationship using linear regression analysis between pain intensity NRS (day 8) as the dependent variable and expectation of pain decrease NRS (day 1) and the following covariates as an independent variable: patients’ characteristics such as age, gender, PS (day 1), genotype of COMT, pain intensity NRS (day 1), and total scores of HADS (day 1). We also tested the association between pain intensity NRS (day 8) and cancer types (pancreatic cancer or not) because pancreatic cancer patients had significantly lower expectation of pain decrease NRS (day1) than the other cancer patients. Then, multiple linear regression analysis was performed using forced entry methods with pain intensity NRS (day 8) as the dependent variable and expectation of pain decrease NRS (day 1) and the covariates as independent variables that had a *p* value <0.05 in simple linear regression analysis, which means that the required level for *p* values was <0.00625 with Bonferroni correction for multiple tests in eight simple regression models. A two-sided significance level of 0.05 was used for multiple regression analysis. All statistical analyses were performed using SPSS software (version 19.0; SPSS Japan Inc., Tokyo).

## Results

### Characteristics of the Patients

The characteristics of the 97 patients (48 males, 49 females) are shown in Table 1. The median age was 69 years (40–85 years). There were 58 inpatients and 39 outpatients. The primary tumors were lung cancer in 39 patients (40%), colorectal cancer in 13, breast cancer in 12, gastric cancer in 8, pancreatic cancer in 5, gallbladder cancer in 4, and cancer of unknown primary cause in 4. More than 90% of the subjects had metastasis and 79% had a PS of 0 to 2. The median required doses of morphine were 30 mg on day 1 (Table 1).

**Table 1:** Clinical characteristics of the patients (*n* = 97)

Characteristics		Number of patients
Age	<65	40
	≥65	57
Gender	Male	48
	Female	49
Performance status (PS)	0–2	77
	3–4	20
Genotype: COMT 472G→A (rs4680, p.Val158Met)	G/G	48
	A/G	37
	A/A	6
	N.E.	6
Tumor types	Lung	39
	Colorectal	13
	Breast	12
	Gastric	8
	Gallbladder	5
	Pancreas	4
	Others	16
Required dose of morphine on day 1	20 mg	5
	30 mg	78
	60 mg	11
	90 mg	2
	N.E.	1
NRS mean (SD)	Day 1 (before treatment)	6.83 (2.22)
	Day 8	3.53 (2.49)
HADS total scores (SD)	Day 1 (before treatment)	15.28 (7.91)
	Day 8	14.89 (7.73)
Expectation of pain decrease	Day 1 (before treatment)	6.70 (2.59)

*N.E.* not evaluated

### Factors Influencing Pain Intensity NRS (Day 8)

With regard to simple regression analysis with pain intensity NRS (day 8) as the dependent variable, only expectation of pain decrease (day 1) and PS (day 1) reached the set *p* value (Table 2). Then, multiple linear regression analysis was performed using forced entry methods with pain intensity NRS (day 8) as the dependent variable, and PS (day 1) and expectation of pain decrease NRS (day 1) as independent variables. The overall fit of the model was examined by using adjusted *R* ^2^ values (0.28) and leverage (0.11).

**Table 2:** Independent determinants of pain on day 8

Item	Simple regression model				Multiple regression model (*R* ^2^ = 0.28)				
	*β*	*t* value	Partial regression coefficient (95% CI)	*p* value	*β*	*t* value	Partial regression coefficient (95% CI)	*p* value	VIF
Age	−0.07	−0.61	−0.17 (−0.07 to 0.04)	0.54					
Gender	0.04	0.36	0.20 (−0.92 to 1.32)	0.72					
Genotype	0.02	0.14	0.08 (−1.04 to 1.20)	0.89					
PS (day 1)	−0.31	−2.89	−1.04 (−1.75 to −0.32)	0.005*	−0.35	−3.40	−1.18 (−1.88 to −0.49)	0.001	1.00
Tumor types	0.01	0.10	0.15 (−2.79 to 3.08	0.92					
HADS (day 1)	0.22	1.96	0.11 (−0.11 to 0.22)	0.05					
Expectation of pain decrease (day 1)	−0.39	−2.78	−0.32 (−0.53 to −0.12)	0.003*	−0.36	−3.50	−0.34 (−0.54 to −0.15)	0.001	1.00
NRS (day 1)	0.21	1.88	0.23 (−0.13 to 0.48)	0.06					

*β* standardized partial regression coefficient, *VIF* variance inflation factors

*p < 0.00625 was statistically significant using Bonferroni correction for multiple tests

Patients with a high expectation of pain decrease NRS on day 1 had a significantly lower pain intensity NRS (day 8) (*p* = 0.001, partial correlation coefficient = 0.36). Those with a low PS also had a significantly lower pain intensity NRS (day 8) (*p* = 0.001) (Table 2). The normality of the residuals of the final model of multiple regression analysis was confirmed using Kolmogorov-Smirnov test while independent variables did not have normal distribution.

Pain intensity NRS (day 8) showed almost no correlation with HADS total score on day 8 (*r* = 0.199).

## Discussion

The results of this study show that patients with an expectation for pain decrease NRS (day 1) had a significantly lower pain intensity NRS (day 8). To our knowledge, this is the first prospective study to examine the effect of an expectation of a change in pain before morphine treatment on pain score 1 week later in opioid-naïve patients with various cancers and PS. The results are consistent with findings in non-cancer patients (whiplash injury, back pain) that pain score is decreased in patients who expect pain improvement [12, 13].

To explain how a health expectation influences the outcome, research on the mechanisms of placebo effects, of which expectancy is generally considered to be the core mechanism, provides more well-grounded suggestions for the causal influence of expectations on pain and other outcomes and for suggestions to improve pain management by modulating expectations [27].

According to the response expectancy theory of Kirsch et al., response expectancy is defined as the anticipation of automatic, subjective, and behavioral responses to particular situational cues, and studies of the effects of placebos reveal that response expectancies can produce lasting changes in pain [28].

An investigation of the effects of the expectation of pain decrease NRS (day 1) indicated some effects on pain intensity NRS (day 8) (partial correlation coefficient = 0.36) in the present study. According to the meta-analyses on placebo and expectancy effects by Vase et al. [29], it was reported that magnitudes of placebo analgesic effects were relatively low (Hedges’ *g* = 0.15) in clinical analgesic trials (studies used only placebo as a control condition) as compared to studies of placebo analgesia mechanisms (Hedges’ *g* = 0.95). Further comparison between acute and chronic pain indicated that magnitudes of placebo analgesic effects have been smaller (Hedges’ *g* = 0.33) in chronic pain than acute procedural pain (Hedges’ *g* = 0.67) in the recent meta-analyses by Peerdeman et al. [30]. Although the measures in previous studies were different and unable to be simply compared due to the difference in study designs, the expectation of pain decrease NRS (day 1) in the present study might have a medium effect size with the partial correlation coefficient. Interplay between symptoms (pain, other symptoms) and pessimistic illness perceptions, an increased focus on bodily sensations, and inexpedient coping strategies should also be investigated in future studies [12].

Our results suggest that pain relief in cancer patients can be improved by increasing the patient’s expectation of pain improvement. However, these findings are from a prospective observational study, and an interventional study is required for further evaluation.

Patients with low PS also had lower pain intensity NRS (day 8) (*p* = 0.001), which may be because most of low PS patients were inpatients who received improved pain control due to more frequent contact with medical staff.

This study has several limitations. First, 20–40% of cancer patients have mental disorders that require therapy [31]. Scores of 11 or greater for the anxiety or depression scale of HADS are the cutoff for such disorders [32], and 67 of the 97 patients in the study (69%) had the anxiety or depression scale of HADS score of ≥11, which is more than in previous studies [31]. This may be because patients with cancer pain were selected as subjects. Second, the patients were included by the decisions of attending physicians in two hospitals only and not continuous sampling; we should consider selection bias, and the results may not be generalizable to other institutions. Third, disease duration, comorbidity, use of drugs other than NSAIDs and opioids, and psychosocial factors including education level and occupation were not examined. Finally, the possibility of response bias as a result of using observer ratings and self-reported questionnaires must be taken into consideration. Several studies have shown that if we ask patients about their expectations of treatment outcome, the expectation scores are very often highly predictive for reports of treatment effectiveness under the influence of biases traditionally termed leniency and halo errors [33, 34]. We should consider this well-known psychological effect. Considering response bias, we attempted to prevent it from impacting our findings in a negative manner; nurses who were not related to our research handed the questionnaire to patients; however, it might be possible that some study results were due to a response bias rather than the hypothesized effect.

Within these limitations, we conclude that cancer patients with pain who expect to have relief from pain before pain control treatment are more likely to have an actual improvement of pain. For control of cancer pain, it may be helpful for oncologists and palliative care doctors to develop their communication skills. By improving the communication skills to improve the doctor-patient relationship, the amount of required dose of analgesics can be reduced by the rise of “expectations” of a patient and the reduction of pain. It may be possible to treat cancer patients of chemical coping [20–22], which has recently become a problem.

## References

[1] Cleeland CS (1998). Undertreatment of cancer pain in elderly patients. JAMA.

[2] Patrick DL, Ferketich SL, Frame PS, Haris JJ, Hendricks CB, Levin B (2004). National Institutes of Health State-of-the-Science Panel. National Institutes of Health State-of-the-Science Conference Statement: symptom management in cancer: pain, depression, and fatigue, July 15-17, 2002. J Natl Cancer Inst Monogr.

[3] Butler LD, Koopman C, Cordova MJ, Garlan RW, DiMiceli S, Spiegel D (2003). Psychological distress and pain significantly increase before death in metastatic breast cancer patients. Psychosomatic Med.

[4] Ger LP, Ho ST, Sun WZ, Wang MS, Cleeland CS (1999). Validation of the Brief Pain Inventory in a Taiwanese population. J Pain Symptom Manag.

[5] Kelson DP, Portenoy RK, Thaler HT, Niedzwiecki D, Passik SD, Tao Y (1995). Pain and depression in patients with newly diagnosed pancreas cancer. J Clin Oncol.

[6] Turk DC, Sist TC, Okifuji A, Miner MF, Florio G, Harrison P (1998). Adaptation to metastatic cancer pain, regional/local cancer pain and non-cancer pain: role of psychological and behavioral factors. Pain.

[7] Wells N, Murphy B, Wujcik D, Johnson R (2003). Pain-related distress and interference with daily life of ambulatory patients with cancer with pain. Oncol Nurs Forum.

[8] Williamson GM, Schulz R (1995). Activity restriction mediates the association between pain and depressed affect: a study of younger and older adult cancer patients. Psychol Aging.

[9] Ahles TA, Blanchard EB, Ruckdeschel JC (1983). The multidimensional nature of cancer-related pain. Pain.

[10] McGuire DB (1992). Comprehensive and multidimensional assessment and measurement of pain. J Pain Symptom Manag.

[11] Quartana PJ, Campbell CM, Edwards RR (2009). Pain catastrophizing: a critical review. Expert Rev Neurother.

[12] Gehrt TB, Wisbech Carstensen TB, Ørnbøl E, Fink PK, Kasch H, Frostholm L (2015). The role of illness perceptions in predicting outcome after acute whiplash trauma: a multicenter 12-month follow-up study. Clin J Pain.

[13] Johansson MS, Boyle E, Hartvigsen J, Jensen Stockkendahl M, Carroll L, Cassidy JD (2015). A population-based, incidence cohort study of mid-back pain after traffic collisions: factors associated with global recovery. Eur J Pain.

[14] Leeuw M, Goossens ME, Linton SJ, Crombez G, Boersma K, Vlaeyen JW (2007). The fear-avoidance model of musculoskeletal pain: current state of scientific evidence. J Behav Med.

[15] Mondloch MV, Cole DC, Frank JW (2001). Does how you do depend on how you think you’ll do? A systematic review of the evidence for a relation between patient’s recovery expectations and health outcomes. CMAJ.

[16] Mahomed NN, Liang MH, Cook EF, Daltroy LH, Fortin PR, Fossel AH, Katz JN (2002). The importance of patient expectations in predicting functional outcomes after total joint arthroplasty. J Rheumatol.

[17] Iles RA, Davidson M, Taylor NF (2008). Psychosocial predictors of failure to return to work in non-chronic non-specific low back pain: a systematic review. Occup Environ Med.

[18] Cheung CW, Qiu Q, Choi SW, Moore B, Goucke R, Irwin M (2014). Chronic opioid therapy for chronic non-cancer pain: a review and comparison of treatment guidelines. Pain Physician.

[19] Chou R, Fanciullo GJ, Fine PG, Adler JA, Ballantyne JC, Davies P (2009). Opioid treatment guidelines. Clinical guidelines for the use of chronic opioid therapy in chronic noncancer pain. J Pain.

[20] Del Fabbro E (2014). Assessment and management of chemical coping in patients with cancer. J Clin Oncol.

[21] Kwon JH, Tanco K, Park JC, Wong A, Seo L, Liu D (2015). Frequency, predictors, and medical record documentation of chemical coping among advanced cancer patients. Oncologist.

[22] Kim YJ, Dev R, Reddy A, Hui D, Tanco K, Park M (2016). Association between tobacco use, symptom expression, and alcohol and illicit drug use in advanced cancer patients. J Pain Symptom Manag.

[23] Keefe FJ, Abernethy AP, Campbell L C. Psychological approaches to understanding and treating disease-related pain. Annu Rev Psychol. 2005;56:601–30. 10.1146/annurev.psych.56.091103.070302 15709948

[24] Cosco TD, Doyle F, Ward M, McGee H (2012). Latent structure of the Hospital Anxiety And Depression Scale: a 10-year systematic review. J Psychosom Res.

[25] Montagna P (2007). Recent advances in the pharmacogenomics of pain and headache. Neurol Sci.

[26] Matsuoka H, Arao T, Makimura C, Takeda M, Kiyota H, Tsurutani J (2012). Expression changes in arrestin β1 and genetic variation in catechol-O-methyltransferase are biomarkers for the response to morphine treatment in cancer patients. Oncol Rep.

[27] Enck P, Bingel U, Schedlowski M, Rief W (2013). The placebo response in medicine: minimize, maximize or personalize?. Nat Rev Drug Discov.

[28] Kirsch I (1997). Response expectancy theory and application: a decennial review. Appl Prev Psychol.

[29] Vase L, Riley JL, Price DD (2002). A comparison of placebo effects in clinical analgesic trials versus studies of placebo analgesia. Pain.

[30] Peerdeman KJ, van Laarhoven AI, Keij SM, Vase L, Rovers MM, Peters ML, Evers AW (2016). Relieving patients’ pain with expectation interventions: a meta-analysis. Pain.

[31] Derogatis LR, Morrow GR, Fetting J, Penman D, Piasetsky S, Schmale AM (1983). The prevalence of psychiatric disorders among cancer patients. JAMA.

[32] Kugaya A, Akechi T, Okuyama T, Okamura H, Uchitomi Y (1998). Screening for psychological distress in Japanese cancer patients. Jpn J Clin Oncol.

[33] Gove WR, Geerken MR (1977). Response bias in surveys of mental health: an empirical investigation. AJS.

[34] Hoyt WT (2000). Rater bias in psychological research: when is it a problem and what can we do about it?. Psychol Methods.

